# Hospital Admissions for Neurodegenerative Diseases during the First Wave of the COVID-19 Pandemic: A Nationwide Cross-Sectional Study from Germany

**DOI:** 10.3390/brainsci11091219

**Published:** 2021-09-15

**Authors:** Raphael Scherbaum, Eun-Hae Kwon, Daniel Richter, Dirk Bartig, Ralf Gold, Christos Krogias, Lars Tönges

**Affiliations:** 1 Department of Neurology, St. Josef-Hospital, Ruhr-University Bochum, 44801 Bochum, Germany; raphael.scherbaum@rub.de (R.S.); eunhae.kwon@rub.de (E.H.K.); daniel.richter-c34@rub.de (D.R.); ralf.gold@rub.de (R.G.); christos.krogias@rub.de (C.K.); 2 DRG MARKET, D-49069 Osnabrück, Germany; dirk.bartig@drg-market.de; 3 Neurodegeneration Research, Protein Research Unit Ruhr (PURE), Ruhr University Bochum, 44801 Bochum, Germany

**Keywords:** Parkinson’s disease, COVID-19, progressive supranuclear palsy (PSP), multiple system atrophy (MSA), amyotrophic lateral sclerosis (ALS)

## Abstract

(1) Background: The COVID-19 pandemic impacts healthcare utilization across all care settings and health conditions. The objective of this study was to determine changes in hospital admissions for neurodegenerative diseases (NDD) during the first COVID-19 wave in Germany; (2) Methods: This cross-sectional study used nationwide administrative claims data covering 1468 hospitals. The primary outcome was the year-to-year relative change in case numbers during a four-month study period (16 January–15 May 2020 vs. 2019) during the first pandemic wave. Secondary outcomes included year-to-year relative changes during a four-week peak phase (16 March–15 April) and changes between differential phases of the wave. The analyzed NDD comprised progressive supranuclear palsy (PSP), multiple system atrophy (MSA), Parkinson’s disease, amyotrophic lateral sclerosis (ALS) and Huntington’s disease; (3) Results: Hospital admissions for any reason decreased by 16.7% in 2020 during the study period and by 36.6% during the peak phase, whereas admissions for NDD decreased by 27.6% and 65.0%, respectively. PSP cases decreased during the study period (−34.7%) and the peak phase (−68.1%) and stayed reduced in a late phase with falling COVID-19 numbers. MSA and ALS cases increased strongest after the peak, with ALS cases being comparatively weakly reduced during the study period (−17.3%) and peak phase (−51.7%); (4) Conclusions: Inpatient care utilization for NDD changed differentially during the first wave of the COVID-19 pandemic in Germany and showed a greater reduction than overall and general neurological admissions. Mitigating long-term health deterioration of this vulnerable subgroup is important to reduce morbidity and mortality in the future.

## 1. Introduction

The WHO declared COVID-19 a pandemic on 11 March 2020 [1]. From March 16 onwards, German hospitals were required to postpone elective inpatient treatments in order to increase intensive care capacities for COVID-19 patients [2]. A lockdown including social distancing measures and reduction of economic and public life started on 22 March and was loosened from 6 May 2020 onwards [3]. As a result, overall hospital admissions declined by 35% during the four weeks after 16 March compared to 2018, as an analysis of 18 German university hospitals has shown [4].

The COVID-19 pandemic thus impacts healthcare delivery and utilization, which has been shown to occur across all care settings and health conditions [5,6] including neurological diseases [7,8]. Rather than by COVID-19 itself, this impact is mediated by responses of society and individuals to COVID-19. These responses comprise containment strategies (e.g., stay-at-home orders, curfews, socioeconomic lockdowns), mitigation strategies of high-level COVID-19 caseload (e.g., postponing non-urgent hospital admissions) and affective and behavioral changes of individuals (e.g., fears of contracting the virus, social distancing measures). Eventually, they might lead to altered healthcare delivery and utilization, e.g., to a decline in hospital admissions for conditions other than COVID-19.

Research on these indirect health effects [5] of the COVID-19 pandemic primarily focused on reduction of overall patient numbers, on urgent medical conditions (e.g., oncological treatments), and on emergencies such as cardio- and cerebrovascular diseases [5,8]. However, chronic neurological conditions, such as neurodegenerative diseases (NDD), also require continued and comprehensive healthcare, including hospital admissions in case of an exacerbation. Accordingly, the management of existing chronic neurological conditions is a key point of the EAN consensus statement for management of patients with neurological diseases during the COVID-19 pandemic [9]. Various publications have shown that the COVID-19 pandemic significantly affects the lives of people living with NDD, for example, amyotrophic lateral sclerosis (ALS) [10,11], multiple system atrophy (MSA) [12] or Parkinson’s disease (PD) [13,14]. For PD, a recent analysis showed reductions of hospital admissions by up to 72.7% during the first pandemic wave [7]. Nevertheless, there is a lack of data on changes in inpatient care utilization for the broad spectrum of further NDD including movement disorders such as MSA and progressive supranuclear palsy (PSP).

Therefore, the objective of this study was to determine changes in the numbers of hospital admissions for various NDD during the first wave of the COVID-19 pandemic in Germany.

## 2. Materials and Methods

**Figure 1:**
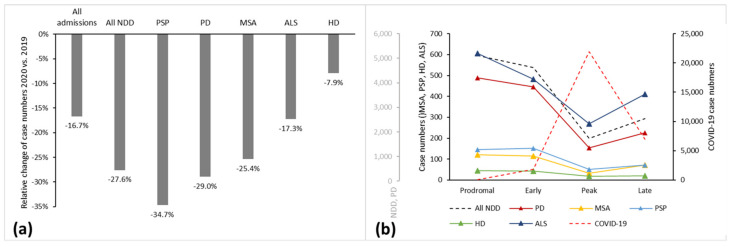
Admission numbers of various neurodegenerative diseases and COVID-19. (**a**) Relative changes 2020 vs. 2019 between January 16 and May 15; (**b**) Absolute count throughout the course of four phases of the first pandemic wave.

We conducted a cross-sectional study using the nationwide administrative claims database covering 1468 hospitals and 11,464,523 patient admissions between 16 January and 15 May 2020 (5,210,432) and 2019 (6,254,091), respectively. The high-quality and validated diagnosis-related group (DRG) database has been described in detail previously for this purpose [7]. No informed consent or ethical approval was required, as this analysis of anonymized secondary data was provided by the German Federal Statistical Office complying with the German data protection regulations. Data were retrieved retrospectively on 19 October 2020. The primary outcome was the year-to-year relative change in inpatient case numbers of NDD patients during a four-month period covering the first pandemic wave (16 January–15 May 2020). The analyzed NDD comprised PSP, MSA, PD, ALS, and Huntington’s disease (HD). Accordingly, inclusion criteria comprised the presence of the respective ICD/DRG codes, whereas any other ICD code served as an exclusion criterion, together with key “06” (discharge to another hospital), thereby avoiding multiple counting. Overall hospital admissions were used as a reference. According to the dynamic of COVID-19 cases [7], we divided the course of the first wave of the pandemic into four phases of a one-month duration each, including a prodromal, an early, a peak, and a late phase with corresponding inpatient numbers of COVID-19 (Figure 1b, Table 1). Secondary outcomes included year-to-year relative changes during the predefined four-week peak phase. Furthermore, we investigated pre/post relative changes between the different phases of the first wave to account for temporal trends. We used the main diagnosis codes according to the International Classification of Diseases 10th revision, German Modification (ICD-10-GM), as shown in Table 1. The statistical analysis was done in a descriptive approach.

## 3. Results

Case numbers of the analyzed NDD and COVID-19 during the analyzed period in 2020 and 2019 as well as relative changes between years and between phases of the first wave are displayed in Table 1.

As shown previously [7], overall hospital admissions decreased by 16.7% during the analyzed four-month period between 16 January and 15 May 2020, compared to 2019 (Figure 1a). Hospital admissions dropped by 36.6% during the four-week peak phase starting on March 16 (Table 1). Patients with NDD were even less frequently admitted in 2020 (full period: −27.6%; peak phase: −65.0%). The subgroup of PSP cases showed the most substantial decline during the full period (−34.7%), while PD (−29.0%) and MSA (−25.4%) admissions decreased within a range around the average of the NDD group. ALS (−17.3%) and HD (−7.9%) decreased less intensely. In general, case numbers of MSA and especially HD were small (<400; Table 1), which is why we focused on PSP and ALS.

During the sharp rise in COVID-19 cases between the early and the peak phase, NDD cases decreased by 63.1% in pre/post comparison, again stronger than overall admissions (−34.2%). By analogy with year-to-year comparison, strong relative changes occurred in PSP (−65.6%) admissions, while ALS (−44.0%) cases decreased less intensely than average. With the substantial drop of COVID-19 cases in the second half of April, i.e., the start of the late phase, all NDD admissions increased again (+11.7% compared to the peak phase). Together with MSA (+125.0%), ALS increased most strongly (+51.9%), whereas PSP (+38.5%) and HD (+10.5%) increased the least.

As an indicator of a more sustained reduction of admissions, relative changes of case numbers between the early and the late phase were high for PSP (−52.3%), whereas ALS cases were reduced least (−14.9%) after the peak phase.

## 4. Discussion

In order to assess the impact of the COVID-19 pandemic on inpatient care of various neurodegenerative diseases during the first wave in Germany, we conducted a cross-sectional study using a high-quality nationwide administrative claims database.

The key findings are that (1) a reduction of inpatient care utilization was pronounced for NDD; (2) PSP cases decreased strongly both during the four-week peak phase and during the entire period, with sustained depression in the late phase with falling COVID-19 case numbers; (3) MSA and ALS cases increased most strongly after the peak, with ALS cases being overall less affected by the reduction of admissions during the first wave of the COVID-19 pandemic.

The decline in overall hospital admissions of 36.6% is in line with a previous finding of 35% from a study of 18 German university hospitals applying a similar period of interest [4]. A meta-analysis including 32 studies on admission changes for different reasons for admission during early pandemic periods reported a median reduction in admissions of 28% compared to pre-pandemic periods [6]. During the peak phase, the decline of admissions was pronounced for NDD compared to diseases of the nervous system in general (−65.0% vs. −51.1%) [4].

The strong and sustained decrease in admissions for PSP could imply a cautious attitude in people living with PSP. For PD, worries and anxiety associated with COVID-19 have been described [14] and represent one possible reason for this finding. It seems unlikely that falls as a reason for admission in PSP occurred less during lockdown. Possibly, a higher proportion of patients with Richardson’s syndrome (PSP-RS) could account for the strong decrease in admissions as this subtype is characterized by a more rapid motor progression than, e.g., the parkinsonism subtype (PSP-P). Yet, the available database does not provide PSP subtype information. As mentioned in the introductory section, stay-at-home orders and reduced inpatient care supply are further reasons for reduced admissions.

The comparatively weaker reduction of admissions for ALS could imply that a high demand for inpatient care outbalanced caution or fears in ALS patients and their caregivers. Common reasons for hospital admission in ALS are respiratory failure, nutritional deficiency, and pneumonia, rendering those admissions mostly non-elective [15].

In order to ensure continued and comprehensive care of people living with NDD and to mitigate health deterioration caused by the pandemic, quick (re-)access to multidisciplinary care and telemedicine play a key role [16], the latter being utilized increasingly since the pandemic’s beginning [17]. In this context, the Bioethics Study Group of the Italian Neurological Society advocates the concept of preparedness when implementing action strategies in public health to be ready for future emergencies caused by pandemics [18].

In the future, both indirect (e.g., changes in hospital care utilization) and direct health effects (e.g., possible causation or exacerbation of NDD) of the COVID-19 pandemic most likely will continue to affect people living with NDD. Even if some studies suggest a causal role of SARS-CoV-2 for neurodegeneration [19], long-term data on COVID-19-related incidence of NDD and conclusive evidence on underlying mechanisms, such as neurodegeneration secondary to microglial neuroinflammation [20], are currently lacking [21,22,23]. Therefore, the question whether or not the COVID-19 pandemic may lead to increased numbers of NDD admissions in the long-term can only be answered by future epidemiological research [21,23]. Limitations of this report include small group sizes, especially for HD and MSA. Additionally, the database does not provide information on reasons for hospital admission. Possible heterogeneities of disease subgroups, such as PSP Richardson’s syndrome or PSP parkinsonism, could not be considered comprehensively. Further studies are needed to study the indirect health effects of COVID-19 on NDD patients and to evaluate the long-term effects of reduced healthcare utilization for patients with chronic neurological conditions, including the risk of higher morbidity and mortality.

The strengths of this analysis are the nationwide range and the first-time description of indirect health effects of the COVID-19 pandemic on various NDD.

To conclude, inpatient care utilization changed differentially across various NDD during the first wave of the COVID-19 pandemic in Germany and showed a greater reduction than overall and general neurological admissions. Mitigating long-term health deterioration is essential to reduce morbidity and mortality in the future.

## Figures and Tables

**Table 1:** Admission numbers of various neurodegenerative diseases and COVID-19 during the study period in 2020 and 2019, year-to-year and pre/post relative changes between phases of the first wave.

Disease	ICD−Code		Case Number					Relative Change		
			Total	Prodromal	Early	Peak	Late	Peak vs. Early	Late vs. Early	Late vs. Peak
			16 Jan−15 May	16 Jan−15 Feb	16 Feb−15 Mar	16 Mar−15 Apr	16 Apr−15 May			
COVID−19	U07.1	2020	30,827	90	1818	21,918	7001	1105.6%	285.1%	−68.1%
All admissions	All ICD	2019	6,254,091	1,719,250	1,509,265	1,540,771	1,484,805	2.1%	−1.6%	−3.6%
		2020	5,210,432	1,657,386	1,485,173	976,590	1,091,283	−34.2%	−26.5%	11.7%
		Diff.	−1,043,659	−61,864	−24,092	−564,181	−393,522			
		Diff. %	−16.7%	−3.6%	−1.6%	−36.6%	−26.5%			
All NDD	G10 + G12.2 + G20 + G23.1 + G23.2 + G23.3	2019	19,232	5239	4568	4856	4569	6.3%	0.0%	−5.9%
		2020	13,920	5102	4609	1699	2510	−63.1%	−45.5%	47.7%
		Diff.	−5312	−137	41	−3157	−2059			
		Diff. %	−27.6%	−2.6%	0.9%	−65.0%	−45.1%			
PD	G20	2019	15,854	4315	3782	3971	3786	5.0%	0.1%	−4.7%
		2020	11,262	4182	3819	1326	1935	−65.3%	−49.3%	45.9%
		Diff.	−4592	−133	37	−2645	−1851			
		Diff. %	−29.0%	−3.1%	1.0%	−66.6%	−48.9%			
MSA	G23.2 + G23.3	2019	457	127	102	120	108	17.6%	5.9%	−10.0%
		2020	341	122	115	32	72	−72.2%	−37.4%	125.0%
		Diff.	−116	−5	13	−88	−36			
		Diff. %	−25.4%	−3.9%	12.7%	−73.3%	−33.3%			
PSP	G23.1	2019	645	187	160	163	135	1.9%	−15.6%	−17.2%
		2020	421	146	151	52	72	−65.6%	−52.3%	38.5%
		Diff.	−224	−41	−9	−111	−63			
		Diff. %	−34.7%	−21.9%	−5.6%	−68.1%	−46.7%			
HD	G10	2019	139	29	31	43	36	38.7%	16.1%	−16.3%
		2020	128	46	42	19	21	−54.8%	−50.0%	10.5%
		Diff.	−11	17	11	−24	−15			
		Diff. %	−7.9%	58.6%	35.5%	−55.8%	−41.7%			
ALS	G12.2	2019	2137	581	493	559	504	13.4%	2.2%	−9.8%
		2020	1768	606	482	270	410	−44.0%	−14.9%	51.9%
		Diff.	−369	25	−11	−289	−94			
		Diff. %	−17.3%	4.3%	−2.2%	−51.7%	−18.7%			

## Data Availability

The data that support the findings of this study are available from the corresponding author upon reasonable request.

## References

[1] World Health Organization WHO Director-General’s Opening Remarks at the Media Briefing on COVID-19, 11 March 2020. https://www.who.int/director-general/speeches/detail/who-director-general-s-opening-remarks-at-the-media-briefing-on-covid-19---11-march-2020.

[2] Press and Information Office of the Federal Government of the Federal Republic of Germany Meeting of the Federal Chancellor with the Heads of Government of the Länder on 12 March 2020 (German). https://www.bundesregierung.de/breg-de/themen/coronavirus/beschluss-zu-corona-1730292.

[3] Press and Information Office of the Federal Government of the Federal Republic of Germany Conference Call between the Chancellor of the Federal Republic of Germany and the Heads of Government of the Länder on May 6, 2020 (German). https://www.bundesregierung.de/breg-de/suche/telefonschaltkonferenz-der-bundeskanzlerin-mit-den-regierungschefinnen-und-regierungschefs-der-laender-am-06-mai-2020-1750988.

[4] Kapsner L.A., Kampf M.O., Seuchter S.A., Gruendner J., Gulden C., Mate S., Mang J.M., Schüttler C., Deppenwiese N., Krause L. (2021). Reduced Rate of Inpatient Hospital Admissions in 18 German University Hospitals During the COVID-19 Lockdown. Front. Public Health.

[5] Roy C.M., Bollman E.B., Carson L.M., Northrop A.J., Jackson E.F., Moresky R.T. (2021). Assessing the indirect effects of COVID-19 on healthcare delivery, utilization and health outcomes: A scoping review. Eur. J. Public Health.

[6] Moynihan R., Sanders S., A Michaleff Z.A., Scott A.M., Clark J., To E.J., Jones M., Kitchener E., Fox M., Johansson M. (2021). Impact of COVID-19 pandemic on utilisation of healthcare services: A systematic review. BMJ Open.

[7] Scherbaum R., Kwon E.H., Richter D., Bartig D., Gold R., Krogias C., Tönges L. (2021). Clinical Profiles and Mortality of COVID-19 Inpatients with Parkinson’s Disease in Germany. Mov. Disord..

[8] Richter D., Eyding J., Weber R., Bartig D., Grau A., Hacke W., Krogias C. (2021). Analysis of Nationwide Stroke Patient Care in Times of COVID-19 Pandemic in Germany. Stroke.

[9] Von Oertzen T.J., Macerollo A., Leone M.A., Beghi E., Crean M., Oztuk S., Bassetti C., Twardzik A., Bereczki D., Di Liberto G. (2020). EAN consensus statement for management of patients with neurological diseases during the COVID-19 pandemic. Eur. J. Neurol..

[10] Hu C., Chen C., Dong X.-P. (2021). Impact of COVID-19 Pandemic on Patients with Neurodegenerative Diseases. Front. Aging Neurosci..

[11] Consonni M., Telesca A., Bella E.D., Bersano E., Lauria G. (2021). Amyotrophic lateral sclerosis patients’ and caregivers’ distress and loneliness during COVID-19 lockdown. J. Neurol..

[12] Cámara A., Compta Y., Pérez-Soriano A., Montagut N., Baixauli M., Maragall L., Ludeña E., de Reyes J.C.L., Peri-Cusi L., Fernández N. (2021). Effects of COVID-19 pandemic and lockdown on people with multiple system atrophy participating in a therapeutic education program. Park. Relat. Disord..

[13] Ferini-Strambi L., Salsone M. (2021). COVID-19 and neurological disorders: Are neurodegenerative or neuroimmunological diseases more vulnerable?. J. Neurol..

[14] Santos-García D., Oreiro M., Pérez P., Fanjul G., González J.M.P., Painceiras M.J.F., Bartolomé C.C., Aymerich L.V., Sancho C.G., Rodrigo M.D.M.C. (2020). Impact of Coronavirus Disease 2019 Pandemic on Parkinson’s Disease: A Cross-Sectional Survey of 568 Spanish Patients. Mov. Disord..

[15] Dubinsky R., Chen J., Lai S.-M. (2006). Trends in hospital utilization and outcome for patients with ALS: Analysis of a large U.S. cohort. Neurology.

[16] De Marchi F., Contaldi E., Magistrelli L., Cantello R., Comi C., Mazzini L. (2021). Telehealth in Neurodegenerative Diseases: Opportunities and Challenges for Patients and Physicians. Brain Sci..

[17] Hassan A., Mari Z., Gatto E.M., Cardozo A., Youn J., Okubadejo N., Bajwa J.A., Shalash A., Fujioka S., Aldaajani Z. (2020). Global Survey on Telemedicine Utilization for Movement Disorders During the COVID-19 Pandemic. Mov. Disord..

[18] Zullo S., Ingravallo F., Crespi V., Cascioli M., D’Alessandro R., Gasperini M., Lalli C., Lugaresi A., Marogna M., Mori M. (2021). The impact of the COVID-19 pandemic on people with neurological disorders: An urgent need to enhance the health care system’s preparedness. Neurol. Sci..

[19] Yang A.C., Kern F., Losada P.M., Agam M.R., Maat C.A., Schmartz G.P., Fehlmann T., Stein J.A., Schaum N., Lee D.P. (2021). Dysregulation of brain and choroid plexus cell types in severe COVID-19. Nature..

[20] Alster P., Madetko N., Koziorowski D., Friedman A. (2020). Microglial Activation and Inflammation as a Factor in the Pathogenesis of Progressive Supranuclear Palsy (PSP). Front. Neurosci..

[21] Bouali-Benazzouz R., Benazzouz A. (2021). Covid-19 Infection and Parkinsonism: Is There a Link?. Mov. Disord..

[22] Merello M., Bhatia K.P., Obeso J.A. (2021). SARS-CoV-2 and the risk of Parkinson’s disease: Facts and fantasy. Lancet Neurol..

[23] Gonzalez-Latapi P., Fearon C., Fasano A., Lang A.E. (2021). Parkinson’s Disease and COVID-19: Do We Need to Be More Patient?. Mov. Disord..

